# Structure of the Recombinant *Neisseria gonorrhoeae* Adhesin Complex Protein (rNg-ACP) and Generation of Murine Antibodies with Bactericidal Activity against Gonococci

**DOI:** 10.1128/mSphere.00331-18

**Published:** 2018-10-10

**Authors:** Hannia Liliana Almonacid-Mendoza, María Victoria Humbert, Aiste Dijokaite, David W. Cleary, Yiwen Soo, Miao-Chiu Hung, Christian M. Orr, Moritz M. Machelett, Ivo Tews, Myron Christodoulides

**Affiliations:** a Neisseria Research Group, Molecular Microbiology, Academic Unit of Clinical and Experimental Sciences, Sir Henry Wellcome Laboratories, University of Southampton Faculty of Medicine, Southampton, United Kingdom; b Antibody and Vaccine Group, Cancer Sciences Unit, University of Southampton Faculty of Medicine, Southampton, United Kingdom; c Hamburg Centre for Ultrafast Imaging & Institute for Nanostructure and Solid State Physics, University of Hamburg, Hamburg, Germany; d Biological Sciences, Institute for Life Sciences, University of Southampton, Southampton, United Kingdom; Food and Drug Administration

**Keywords:** NGO1981, *Neisseria gonorrhoeae*, adhesin complex protein, bactericidal antibody, crystal structure, recombinant protein production, vaccine

## Abstract

Neisseria gonorrhoeae (gonococcus [Ng]) is the causative organism of the sexually transmitted disease gonorrhoea, and no effective vaccine exists currently. In this study, the structure, biological properties, and vaccine potential of the Ng-adhesin complex protein (Ng-ACP) are presented. The crystal structure of recombinant Ng-ACP (rNg-ACP) protein was solved at 1.65 Å. Diversity and conservation of Ng-ACP were examined in different *Neisseria* species and gonococcal isolates (https://pubmlst.org/neisseria/ database) *in silico*, and protein expression among 50 gonococcal strains in the Centers for Disease Control and Prevention/Food and Drug Administration (CDCP/FDA) AR Isolate Bank was examined by Western blotting. Murine antisera were raised to allele 10 (strain P9-17)-encoded rNg-ACP protein with different adjuvants and examined by enzyme-linked immunosorbent assay (ELISA), Western blotting, and a human serum bactericidal assay. Rabbit antiserum to rNg-ACP was tested for its ability to prevent Ng-ACP from inhibiting human lysozyme activity *in vitro.* Ng-ACP is structurally homologous to Neisseria meningitidis ACP and MliC/PliC lysozyme inhibitors. Gonococci expressed predominantly allele 10- and allele 6-encoded Ng-ACP (81% and 15% of isolates, respectively). Murine antisera were bactericidal (titers of 64 to 512, *P* < 0.05) for the homologous P9-17 strain and heterologous (allele 6) FA1090 strain. Rabbit anti-rNg-ACP serum prevented Ng-ACP from inhibiting human lysozyme with ∼100% efficiency. Ng-ACP protein was expressed by all 50 gonococcal isolates examined with minor differences in the relative levels of expression. rNg-ACP is a potential vaccine candidate that induces antibodies that (i) are bactericidal and (ii) prevent the gonococcus from inhibiting the lytic activity of an innate defense molecule.

**IMPORTANCE** Neisseria gonorrhoeae (gonococcus [Ng]) is the causative organism of the sexually transmitted disease gonorrhoea, and the organism is listed by the World Health Organization as a high-priority pathogen for research and development of new control measures, including vaccines. In this study, we demonstrated that the N. gonorrhoeae adhesin complex protein (Ng-ACP) was conserved and expressed by 50 gonococcal strains and that recombinant proteins induced antibodies in mice that killed the bacteria *in vitro*. We determined the structure of Ng-ACP by X-ray crystallography and investigated structural conservation with Neisseria meningitidis ACP and MliC/PliC proteins from other bacteria which act as inhibitors of the human innate defense molecule lysozyme. These findings are important and suggest that Ng-ACP could provide a potential dual target for tackling gonococcal infections.

## Abstract (precis)

Neisseria gonorrhoeae (gonococcus [Ng]) is the causative organism of the sexually transmitted disease gonorrhoea, and the organism is listed by the World Health Organization as a high-priority pathogen for research and development of new control measures, including vaccines. In this study, we demonstrated that the N. gonorrhoeae adhesin complex protein (Ng-ACP) was conserved and expressed by 50 gonococcal strains and that recombinant proteins induced antibodies in mice that killed the bacteria *in vitro*. We determined the structure of Ng-ACP by X-ray crystallography and investigated structural conservation with Neisseria meningitidis ACP and MliC/PliC proteins from other bacteria which act as inhibitors of the human innate defense molecule lysozyme. These findings are important and suggest that Ng-ACP could provide a potential dual target for tackling gonococcal infections.

## INTRODUCTION

Neisseria gonorrhoeae (gonococcus [Ng]) is the causative organism of the sexually transmitted disease gonorrhoea. Gonococci infect the mucosal epithelium of the genitourinary tract; in men, infection of the urethra causes urethritis and painful discharge, and in women, localized infection of the ectocervix and endocervix leads to a mucopurulent cervicitis. However, gonococcal infection is frequently asymptomatic, and in approximately 10% to 25% of untreated women, the bacteria can ascend into the upper reproductive tract. The host response to this ascending infection is pelvic inflammatory disease (PID), which is marked by severe inflammation, e.g., endometritis, pelvic peritonitis (tubal, ovarian), and salpingitis in the fallopian tubes, and by long-term and/or permanent sequelae, including chronic pelvic pain, tubal damage, ectopic pregnancy, and infertility (1). Gonococci can also cause anorectal and pharyngeal infections and, more rarely, disseminated infection, which can present as arthritis, perihepatitis, meningitis, or endocarditis (2). Infection is particularly severe in neonates, with ophthalmia neonatorum (neonatal conjunctivitis) as the most common manifestation (2, 3). In addition, there is a strong association between maternal gonorrhoea and premature delivery and low neonatal birth weight (4). Gonococcal meningitis and sepsis have been reported, though these are rarer. There are an estimated 78 million cases of gonococcal infection annually worldwide [http://www.who.int/en/news-room/fact-sheets/detail/sexually-transmitted-infections-(stis)] (5), and treatment has relied on antibiotics, but this is being compromised by the emergence of multiantibiotic-resistant gonococci (6). N. gonorrhoeae is listed by the World Health Organization as a high-priority pathogen for research and development of new control measures, including new antimicrobials and vaccines (7, 8).

The goal of an effective preventative gonococcal vaccine has been elusive, and the few vaccines that have entered into clinical trials have largely failed (5). These vaccines included killed whole cells (9), a purified single-antigen pilus-based vaccine (10–12), and porin B isolated from the gonococcus (but which was contaminated with lipooligosaccharide [LOS], Rmp, and Opa protein) (13). Since those trials, a comprehensive list has been collated of potential vaccine antigens that induced bactericidal antibody (Ab) responses in animals (5), but none have progressed to clinical trials. One potential antigen is the adhesin complex protein (ACP, NEIS2075), which has been described in N. meningitidis (meningococcus [Nm]; Nm-ACP/NMB2095), with homologue proteins present in N. gonorrhoeae (Ng-ACP/NGO1981), N. lactamica, and all other *Neisseria* spp. (14). Here, we demonstrated that Nm-ACP was surface exposed, that a recombinant protein induced murine antibodies that killed meningococci via a complement-dependent mechanism, and that the bactericidal response was cross-protective against all the allelic variants found among meningococci (14). The crystal structure of Nm-ACP that we reported earlier (15) revealed structural similarity to the members of the MliC/PliC protein family of membrane-bound or periplasmic inhibitors of human C-type lysozyme (HL). ACP proteins expressed by meningococci, gonococci, and commensal *Neisseria* species all inhibited HL *in vitro,* and bacterial expression conferred tolerance to HL *in vivo*, despite Nm-ACP not sharing the conserved MliC/PliC sequence motifs required for lysozyme recognition (15).

The main objectives of the current study were (i) to solve the structure of Ng-ACP and compare it with the structures of Nm-ACP and HL binding MliC/PliC, (ii) to examine the expression of Ng-ACP among the members of a panel of gonococcal strains, and (iii) to test the hypothesis that recombinant Ng-ACP (rNg-ACP) protein could induce bactericidal antibodies for gonococci expressing the major allelic variant proteins.

(Part of this study was presented at the 20th International Pathogenic *Neisseria* Conference, Manchester, United Kingdom, 4 to 9 September 2016 [16].)

## RESULTS

### Crystal structure of Ng-ACP.

**FIG 1:**
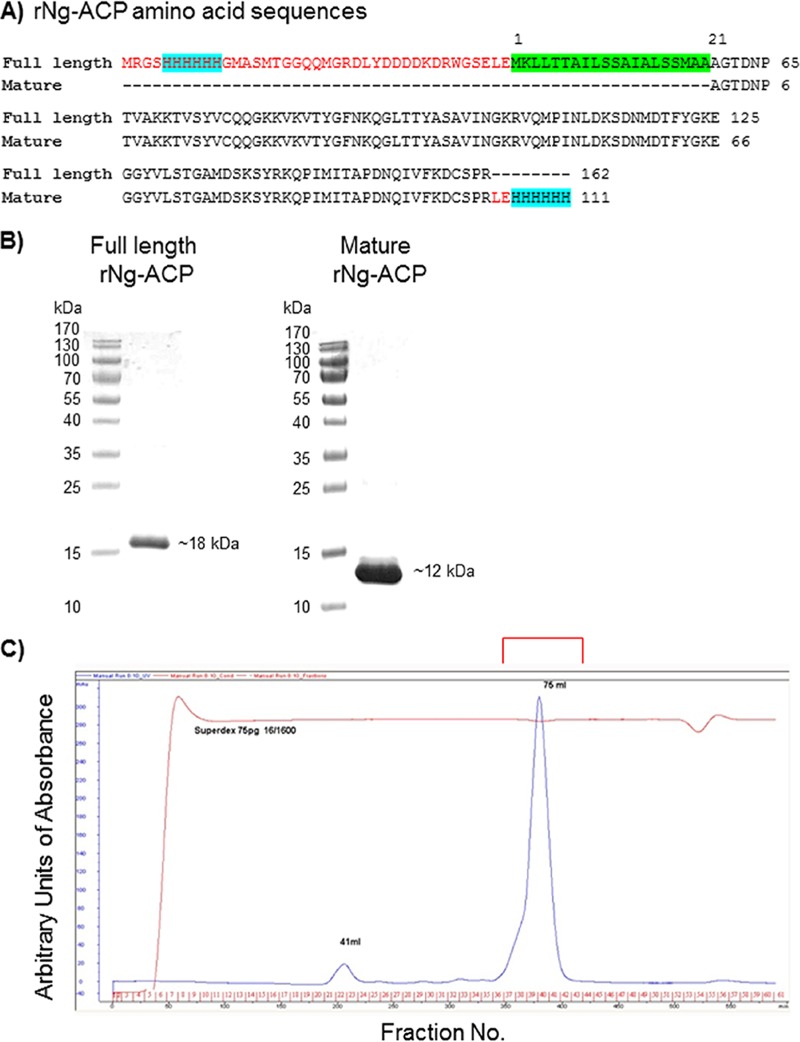
(A) Amino acid sequences for full-length and mature rNg-ACP proteins. The sequences highlighted in red are encoded by the vector, the turquoise sequence is the 6×His tag for purification by affinity chromatography, and the green sequence is the leader peptide sequence. (B) SDS-PAGE analysis of full-length and mature rNg-ACP proteins. Single bands with purity of >95% are shown for full-length (*M*_r_ = ∼18,000) and mature (*M*_r_ = ∼12,000) rNg-ACP proteins. (C) SEC chromatogram of purified mature rNg-ACP, showing a single major peak of protein for crystallography (*M*_r_ = ∼12,000).

We expressed and purified rNg-ACP in Escherichia coli in two variants. Similarly to our study that examined the immunogenicity of rNm-ACP (14), we expressed rNg-ACP with the native N terminus containing the protein leader sequence (amino acids 1 to 21 and a fused 6×His tag). This protein was expressed for immunogenicity studies as a full-length insoluble protein of 162 amino acids with predicted a *M*_r_ of 17,783.18 and was purified under denaturing conditions by the use of nickel-nitriloacetic acid (Ni-NTA) affinity chromatography, followed by solubilization in 0.5% SDS (Fig. 1A and B). A second expression construct that lacked the N-terminal signal peptide sequence and instead contained a C-terminal 6×His tag had a predicted length of 111 amino acids with a *M*_r_ of 12,372.05 (Fig. 1A). The latter construct was used to purify rNg-ACP by Ni-NTA affinity chromatography and size exclusion chromatography (SEC) in soluble form (Fig. 1) for crystallization and structure determination and also immunogenicity studies.

**FIG 2:**
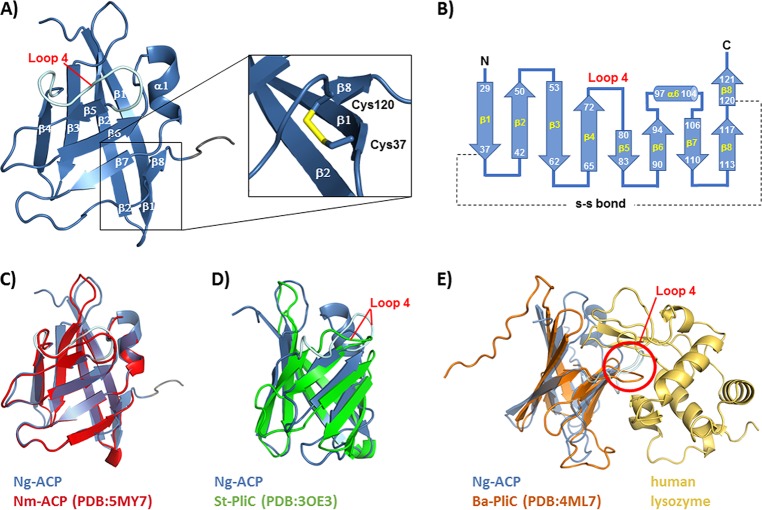
Structure of rNg-ACP. (A) Cartoon representation of the three-dimensional structure of rNg-ACP, showing the antiparallel arrangement of eight β-strands. The position of the stabilizing disulfide bond is shown in stick representation in the zoomed view. (B) Topology diagram showing residue ranges in each β-strand and the -S-S- bond between Cys37 and Cys120 residues. (C) Superposition of Ng-ACP (PDB code 6GQ4, blue) with Nm-ACP (PDB code 5MY7, red). (D) Superposition of Ng-ACP (PDB code 6GQ4, blue) with S. enterica Typhimurium PliC (St-PliC; PDB code 3OE3, green). (E) Superposition of Ng-ACP (PDB code 6GQ4, blue) with the Brucella abortus PliC (Ba-PliC; PDB code 4ML7, orange)-lysozyme complex (gold); the position of loop 4 is indicated by the red circle. Visualization of structures was done with PyMOL.

Crystallization conditions were identified with the Pact *premier* screen and optimized using custom screens. Crystal diffraction data were collected to 1.65 Å, followed by structure determination by molecular replacement using Salmonella enterica serovar Typhimurium PliC (Protein Data Bank [PDB] code 3OE3) as the search model. Data collection and refinement statistics are given in Table 1. The overall fold of rNg-ACP was an eight-stranded β-barrel, and the structure was comprised of two sets of four stranded antiparallel β-sheets (β1 to β4 and β5 to β8), with the disulfide bridge between Cys37 and Cys120 stabilizing the interaction between β8 and β1 (Fig. 2A and B).

**TABLE 1:** Crystal structure of rNg-ACP: data collection and refinement statistics^*a*^

Parameter	Result(s)
Data collection statistics	
Space group	C121
Unit cell parameters	
a, b, c	49.1 Å, 31.0 Å, 67.6 Å
α, β, γ	90°, 103.5°, 90°
X-ray source and wavelength	ID23-1–11.562 keV (1.0723 Å)
Resolution range (Å)	32.86–1.65 (1.68–1.65)
Multiplicity	7.6 (7.7)
I/sigma(I)	14.4 (6.4)
CC1/2	0.993 (0.987)
Total no. of reflections	91,765 (4567)
No. of unique reflections	12,098 (593)
Completeness (%)	99.9 (99.8)
*R merge*	0.088 (0.170)
*R p.i.m.*	0.034 (0.066)
	
Refinement statistics	
No. of protein residues	102
*Rwork*	0.125
*Rfree* (5.0% data)	0.179
No. of nonhydrogen atoms	1,645
Mean overall B (Å)	25.5
RMSD from overall values	
Bond distance (Å)	0.0148
Bond angle (degrees)	1.783
Ramachandran (fav/acc/out)	(84/2/0)
PDB accession code	6GQ4

a Values for the high-resolution shell are shown in parentheses in the second column.

The Dali webserver (17) was used to identify structural homologues in the Protein Data Bank (PDB). Ng-ACP was superposed with Nm-ACP (Fig. 2C) (15) and with members of the MliC/PliC family lysozyme inhibitors found in other Gram-negative bacteria, e.g., Salmonella Typhimurium (St-PliC, Fig. 2D) and Brucella abortus (PliC, Fig. 2E). The structures of B. abortus PliC lysozyme complex (PDB code 4ML7) overlaid the Pseudomonas aeruginosa MliC lysozyme complex (PDB code 3F6Z) well, with a root mean square deviation (RMSD) value of 1.457 (not shown). The superposition of Ng-ACP and the Brucella abortus PliC-lysozyme complex (Fig. 2E) showed a conformation of “loop 4” incompatible with lysozyme binding; furthermore, helix α1 of Ng-ACP would also clash with lysozyme. Our earlier study (15) highlighted these steric clashes for the very similar protein Nm-ACP (compare Fig. 2C) and used a modeling approach that suggested that interactions would require a repositioning of lysozyme or, alternatively, structural changes on Nm-ACP.

**FIG 3:**
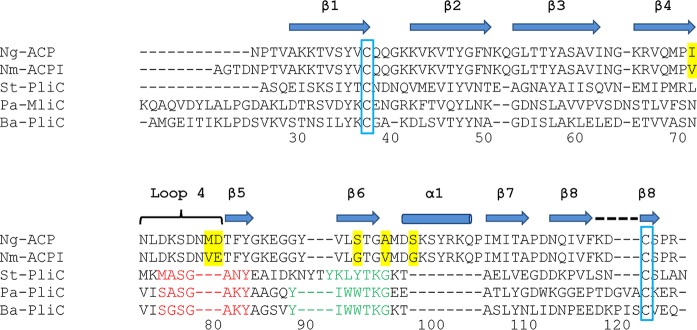
Structure-based sequence alignment of Ng-ACP with Nm-ACP and the MliC/PliC family of lysozyme inhibitor proteins. The structure-based sequence alignment for N. meningitidis serogroup B strain MC58 (E6MZT7), St-PliC from S. enterica Typhimurium (Q8ZPY8), Pa-MliC from Pseudomonas aeruginosa (Q91574), and Ba-PliC from Brucella abortus (Q57ES7) described by Humbert et al. (15) was expanded with the addition of the sequence of Ng-ACP from Neisseria gonorrhoeae P9-17. β-Strands (including β-strand 1 to β-strand 8) are indicated by the shaded arrows and helix α1 by the shaded barrel; conserved cysteine residues of the mature proteins are blocked in blue and sequence motifs involved in the interaction of MliC (P. aeruginosa) with hen egg white lysozyme are indicated in red and green, as described previously (15). Amino acid residues highlighted in yellow show the differences between Ng-ACP and Nm-ACP type I sequences.

**FIG 4:**
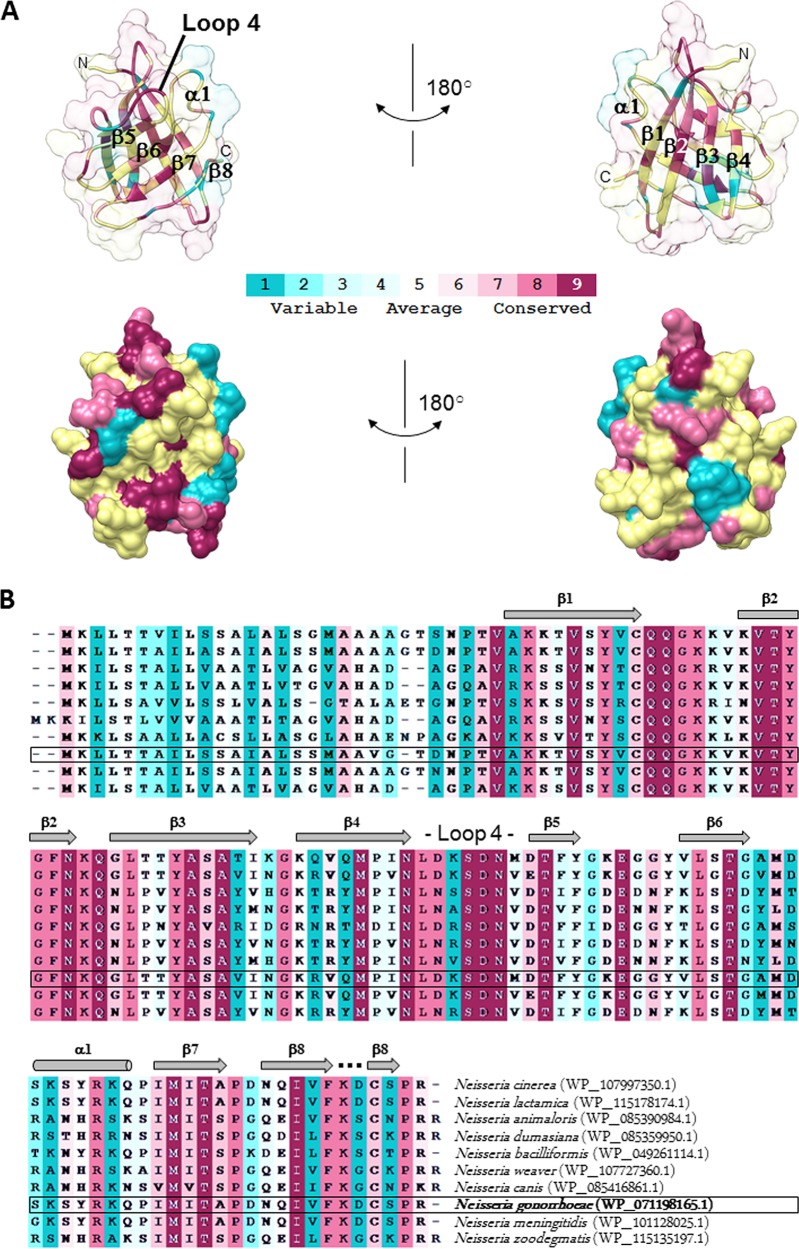
Structure conservation mapping of ACP proteins from different *Neisseria* species. The conservation was calculated by ConSurf (18) and is color coded as shown in the scale from blue (variable) to red (conserved), where average regions are shown in white (compare methods). (A) Structure of Ng-ACP in the same orientation as seen in Fig. 2, shown as a cartoon with transparent surface (top) and as surface (bottom) in two orientations rotated by 180 degrees around the vertical axis in the paper plane. The conservation mapping is based on the sequence alignment shown in panel B.

Ng-ACP, like Nm-ACP, lacks the conserved MliC/PliC sequence motifs involved in interactions with human lysozyme (highlighted in red and green in the structural alignment shown in Fig. 3). The differences in sequence and clashes revealed in the structural superposition have led to the suggestion of the presence of different inhibition mechanisms employed by these two groups of proteins (15). We investigated this further and provide a conservation analysis that is based on sequence alignment of ACP proteins from different *Neisseria* species (Fig. 4). Interestingly, there is little conservation in the region around β6, suggesting that this region is not involved in formation of a conserved interface (compare the green region in Fig. 3). While our analysis revealed some conservation in loop 4 (Fig. 4), this loop was much shorter in the MliC/PliC family of proteins (Fig. 3), explaining why the loop clashed with lysozyme in the superposition (Fig. 2E).

### Conservation and expression of Ng-ACP in Neisseria gonorrhoeae.

**FIG 5:**
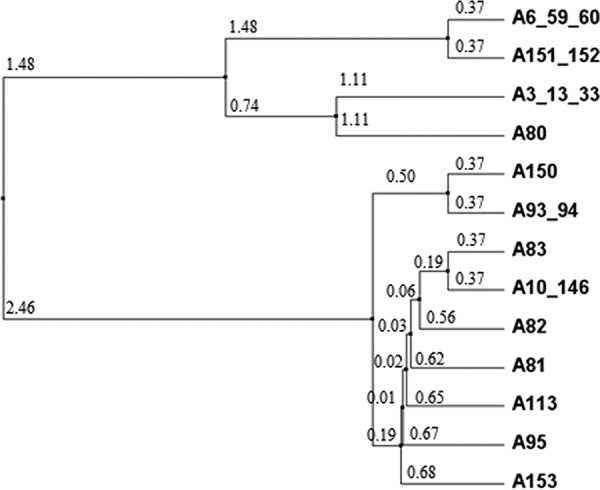
Dendrogram showing the relationships among the 13 nonredundant NEIS2075/NGO1981 alleles in Neisseria gonorrhoeae isolates in the https://pubmlst.org/neisseria/ database. A, allele. Where two or more allele numbers are given, these represent encoded proteins with the same amino acid sequences. The numbers in smaller font denote the average distance using a percent identity tree calculated using Jalview 2.8 (www.jalview.org).

**FIG 6:**
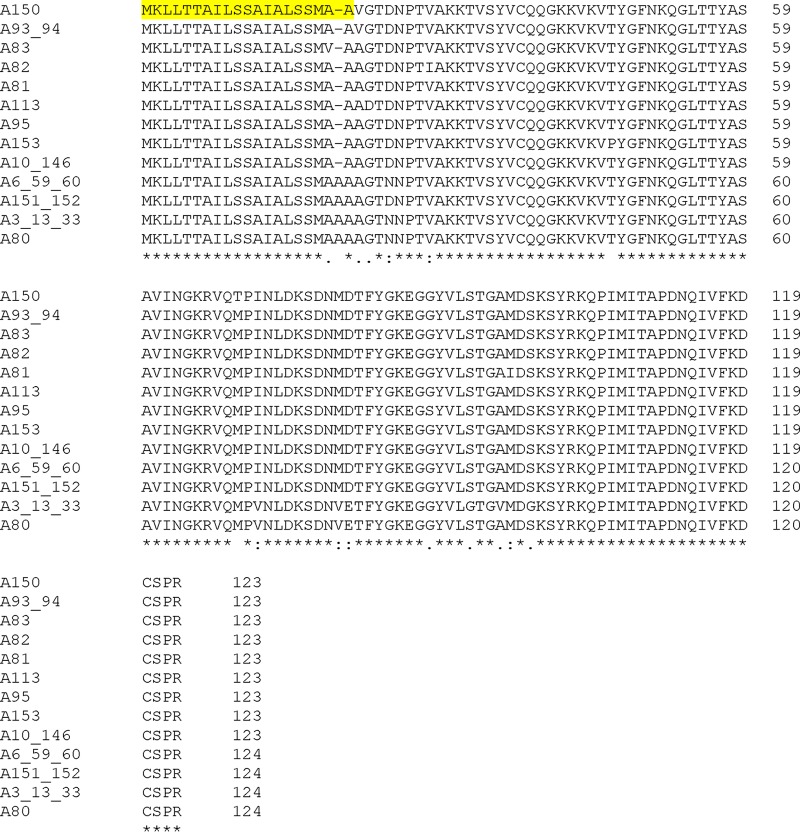
Clustal alignment of amino acid sequences encoded by nonredundant NEIS2075/NGO1981 (Ng-ACP) alleles in the https://pubmlst.org/bigsdb?db=pubmlst_neisseria_isolates database. The database was accessed 4 April 2018. Amino acid sequence alignments were generated using Clustal (http://www.ebi.ac.uk/Tools/msa/clustalo/). A, allele. Asterisks (*) denote fully conserved amino acid residues; colons (:) indicate conservation between groups of strongly similar properties; periods (.) denote conservation between groups of weakly similar properties. Yellow highlighting denotes leader sequence amino acids.

The DNA sequences of the *acp* gene (NEIS2075/NGO1981) from gonococcal isolates in the https://pubmlst.org/neisseria/ database (19) were translated into amino acid sequences and aligned. Among a combined total of 3,876 N. gonorrhoeae isolates in the database with defined alleles, there were 20 different allelic loci for which isolates were identified. An additional 1,041 gonococcal isolates were present with no allele defined, and use of a genome comparator identified an additional 3 isolates (identifiers [ID] 48522, 48823, and 53813) with incomplete sequences. Analysis of the translated proteins encoded by the nucleotide sequences of the 20 different alleles and generation of a dendrogram identified 13 nonredundant NEIS2075 amino acid sequences (Fig. 5). In the https://pubmlst.org/neisseria/ database, 81% (3,128/3,876) of N. gonorrhoeae strains expressed Ng-ACP encoded by allele 10 and 15% (570/3,876) expressed protein encoded by allele 6 (Table 2). Clustal alignment of all 13 nonredundant Ng-ACP protein sequences showed a high degree of amino acid sequence conservation within the mature proteins (amino acids 22 to 124) (Fig. 6). The Ng-ACP proteins encoded by alleles 10 and 6 were ∼98% identical, with a single amino acid substitution (Asp25 to Asn25) and a deletion of Ala20 in the Ng-ACP protein encoded by allele 10. These changes cannot be visualized in the structure as they occurred in the leader peptide that is cleaved off in the mature protein.

**TABLE 2:** Analysis of Ng-ACP (NEIS2075/NGO1981) alleles and number of *Neisseria gonorrhoeae* isolates per allele grouping^*a*^

Allele	No. of isolates	% of total
3 (+13 + 33)	7	0.18
6 (+59 + 60)	570	14.71
10 (+146)	3,128	80.70
80	2	0.05
81	3	0.08
82	5	0.13
83	1	0.03
93 (+94)	136	3.51
95	2	0.05
113	11	0.28
150	6	0.15
151 (+152)	4	0.10
153	1	0.03
		
Total (13 alleles)	3,876	100

a Data were collated from https://pubmlst.org/bigsdb?db=pubmlst_neisseria_isolates. The numbers in parentheses indicate that those alleles produce proteins with identical amino acid sequences. Data were accessed on 4 April 2018; there are 20 allelic loci, with isolates generating 13 nonredundant protein amino acid sequences. The table is sorted in numerical order of allele designations.

**FIG 7:**
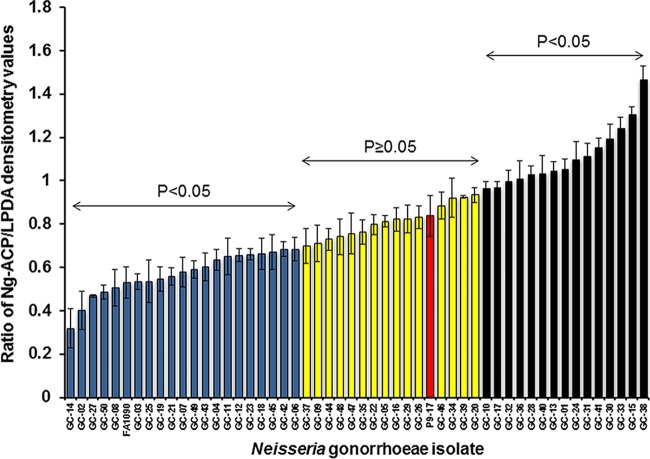
Expression of Ng-ACP protein in the CDCP/FDA N. gonorrhoeae isolate panel. Bacterial lysates were reacted with rabbit anti-ACP serum in Western blots, and the level of Ng-ACP in each lysate was expressed as a ratio of the densitometry of band intensity compared to LPDA. Statistical differences in ratio values were analyzed for each isolate against P9-17 as a reference, which resulted in distribution of the isolates into three main groups, i.e., lower than P9-17 (*P* < 0.05) (blue columns), similar to P9-17 (*P* ≥ 0.05) (yellow columns), and higher than P9-17 (*P* < 0.05) (black columns) Ng-ACP levels. Columns represent means of results from a minimum of three independent measurements of densitometry, and the error bars represent the standard deviations.

To investigate Ng-ACP protein expression among different gonococcal strains, individual bacterial lysates were prepared from the CDCP/FDA AR Isolate Bank of 50 N. gonorrhoeae isolates. Of the 50 isolates, 44 (88%) expressed allele 10-encoded Ng-ACP protein and 6 (12%) expressed allele 6-encoded protein (Table 3). The lysates were reacted with rabbit anti-rACP serum in Western blots, and the level of Ng-ACP in each lysate was expressed as a ratio of the densitometry of band intensity compared to conserved gonococcal dihydrolipoyl dehydrogenase (LPDA) protein as a loading control (Fig. 7). Statistical differences in ratio values were analyzed for each isolate against P9-17 as the standard, which resulted in distribution of the isolates into three main groups, i.e., lower than P9-17 levels, similar to P9-17 levels, and higher than P9-17 Ng-ACP levels (Fig. 7; see also Table 3). There were 15 isolates (30% of the total isolate number) that expressed Ng-ACP at levels higher than P9-17 (*P* < 0.05). Those isolates included all 6 expressing allele 6-encoded protein; 15 isolates (30% of the total isolate number) that expressed Ng-ACP protein encoded by allele 10 at levels similar to P9-17 levels (*P* ≥ 0.05); and 20 isolates (40% of the total isolate number) that expressed Ng-ACP protein encoded by allele 10 at levels lower than the P9-17 levels (*P* < 0.05) (Fig. 7; see also Table 3). Examination of the range of Ng-ACP/LPDA ratios for the isolates showed that there was an ∼4-fold difference in Ng-ACP expression between the highest values (isolate GC-38) and the lowest values (isolate GC-14) determined. In addition, the FA1090 Ng-ACP/LPDA ratio was ∼1.5 fold lower than the P9-17 Ng-ACP/LPDA ratio (Fig. 7).

**TABLE 3:** Determination of Ng-ACP allele in isolates of the *Neisseria gonorrhoeae* CDCP/FDA collection^*a*^

AR-Bank no.	GC no.	Sanger run accession no.	Sanger strain no.	Pubmlst/ *Neisseria* ID	Ng-ACP allele	Level of Ng-ACP protein expression relative to P9-17
165	GC-01	ERR854938	12CFX_T_043	37079	10	High
166	GC-02	ERR854921	12CFX_T_026	37062	10	Low
167	GC-03	ERR855352	12AZI_T_002	37507	10	Low
168	GC-04	ERR854924	12CFX_T_029	37065	10	Low
169	GC-05	ERR854922	12CFX_T_027	37063	10	Similar
170	GC-06	ERR854897	12CFX_T_001	37037	10	Low
171	GC-07	ERR854937	12CFX_T_042	37078	10	Low
172	GC-08	ERR854906	12CFX_T_010	37046	10	Low
173	GC-09	ERR854907	12CFX_T_011	37047	10	Similar
174	GC-10	ERR854900	12CFX_T_004	37040	10	High
175	GC-11	ERR855365	12AZI_T_015	37520	10	Low
176	GC-12	ERR854869	12CFX_T_045	37081	10	Low
177	GC-13	ERR855355	12AZI_T_005	37510	6	High
178	GC-14	ERR855325	12AZI_C_010	37480	10	Low
179	GC-15	ERR855356	12AZI_T_006	37511	6	High
180	GC-16	ERR854913	12CFX_T_017	37053	10	Similar
181	GC-17	ERR855357	12AZI_T_007	37512	6	High
182	GC-18	ERR854870	12CFX_T_047	37083	10	Low
183	GC-19	ERR854902	12CFX_T_006	37042	10	Low
184	GC-20	ERR854916	12CFX_T_020	37056	10	Similar
185	GC-21	ERR854898	12CFX_T_002	37038	10	Low
186	GC-22	ERR854919	12CFX_T_023	37059	10	Similar
187	GC-23	ERR855360	12AZI_T_010	37515	10	Low
188	GC-24	ERR854932	12CFX_T_037	37073	10	High
189	GC-25	ERR854903	12CFX_T_007	37043	10	Low
190	GC-26	ERR854936	12CFX_T_041	37077	10	Similar
191	GC-27	ERR854899	12CFX_T_003	37039	10	Low
192	GC-28	ERR854917	12CFX_T_021	37057	10	High
193	GC-29	ERR855351	12AZI_T_001	37506	10	Similar
194	GC-30	ERR854905	12CFX_T_009	37045	10	High
195	GC-31	ERR854928	12CFX_T_033	37069	10	High
196	GC-32	ERR854927	12CFX_T_032	37068	6	High
197	GC-33	ERR855353	12AZI_T_003	37508	6	High
198	GC-34	ERR854904	12CFX_T_008	37044	10	Similar
199	GC-35	ERR855358	12AZI_T_008	37513	10	Similar
200	GC-36	ERR854929	12CFX_T_034	37070	10	High
201	GC-37	ERR854912	12CFX_T_016	37052	10	Similar
202	GC-38	ERR855359	12AZI_T_009	37514	6	High
203	GC-39	ERR854908	12CFX_T_012	37048	10	Similar
204	GC-40	ERR854867	12CFX_T_024	37060	10	High
205	GC-41	ERR956689	12CFX_T_039	58867	10	High
206	GC-42	ERR956690	12CFX_T_030	58866	10	Low
207	GC-43	ERR855324	12AZI_C_009	37479	10	Low
208	GC-44	ERR854930	12CFX_T_035	37071	10	Similar
209	GC-45	ERR854933	12CFX_T_038	37074	10	Low
210	GC-46	ERR854911	12CFX_T_015	37051	10	Similar
211	GC-47	ERR854910	12CFX_T_014	37050	10	Similar
212	GC-48	ERR854939	12CFX_T_046	37082	10	Similar
213	GC-49	ERR854920	12CFX_T_025	37061	10	Low
214	GC-50	ERR854935	12CFX_T_040	37076	10	Low

a The sequence accession number (https://www.cdc.gov/drugresistance/resistance-bank/currently-available.html) for each isolate is linked to the SRA webpage (https://www.ncbi.nlm.nih.gov/sra/?term=SAMEA3165293) that provides the run accession number that can be used to search the Sanger repository (http://www.sanger.ac.uk/resources/downloads/bacteria/neisseria.html#project_3416) that can identify the strain information. The strain information is then used to search the pubmlst/*Neisseria* webpage (https://pubmlst.org/bigsdb?db=pubmlst_neisseria_isolates&page=query) to identify the ID number, amino acid sequence, and encoding allele. Statistical differences in ratio values were analyzed for each isolate against P9-17 as a reference, which resulted in distribution of the isolates into three main groups, i.e., lower than P9-17 (*P* < 0.05), similar to P9-17 (*P* ≥ 0.05), and higher than P9-17 (*P* < 0.05) Ng-ACP levels.

**FIG 8:**
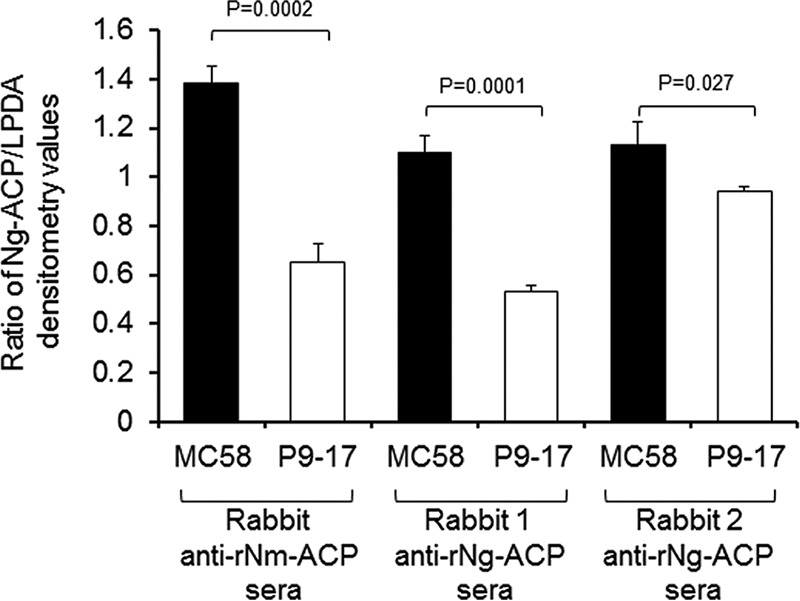
Western blot analysis of expression of ACP in outer membranes (OM) of N. meningitidis strain MC58 and N. gonorrhoeae strain P9-17. OM of N. meningitidis strain MC58 and N. gonorrhoeae strain P9-17 were probed independently with rabbit antisera to rNg-ACP (rabbit 1 and rabbit 2) and with cross-reacting antibody to rNm-ACP in Western blotting, and protein levels were expressed as ratios of the densitometry of band intensity compared to a constitutively expressed dihydrolipoyl dehydrogenase (LPDA) OM protein. The columns represent the mean ratios of results from *n* = 3 experiments with the rabbit antisera to rNg-ACP and *n* = 3 experiments with the rabbit antisera to rNm-ACP and the error bars the standard deviations. Data were analyzed by two-sample *t* test (assuming equal variances), with *P* values of <0.05 denoting significance.

Western blotting was also used to compare the levels of ACP in outer membranes (OM) of N. gonorrhoeae strain P9-17 with N. meningitidis strain MC58 levels, relative to constitutively expressed LPDA. OM from both strains were reacted with antisera from two rabbits immunized with rNg-ACP and also with a rabbit antibody to rNm-ACP (15) that cross-reacted with Ng-ACP (Fig. 8). With the species-specific antisera, there were significantly (*P* < 0.05) lower (∼20% to 50%) levels of Ng-ACP present in P9-17 OM than in MC58 OM, and with the cross-reacting rNm-ACP serum, this difference was ∼53% (*P* < 0.05) (Fig. 8).

### rNg-ACP proteins are antigenic and elicit bactericidal antibodies.

Groups of mice were immunized with purified full-length and mature rNg-ACP proteins (Fig. 1A) with a variety of adjuvant and delivery vehicles, which can enhance the production of antibodies to many other *Neisseria* recombinant proteins, as we have shown in previous studies. rNg-ACP proteins were incorporated into liposomes, which have been used in humans extensively as a nontoxic delivery vehicle for drugs and other molecules and are increasingly present in licensed and experimental human vaccines (20). Liposomes provide an intrinsic adjuvant effect and can permit the folding of proteins, including rNm-ACP (14) and other recombinant OM proteins (21–30). rNg-ACP proteins were also solubilized with the zwitterionic detergent Zwittergent (ZW) 3-14, which provides a micellar structure for protein delivery (31). In an attempt to increase the immunogenicity of the rNg-ACP proteins, we also prepared rNg-ACP liposomes and rNg-ACP-ZW 3-14 micelles that contained the immunomodulator monophosphoryl lipid A (MPLA). MPLA is an adjuvant that is increasingly being used with human vaccines (32), and we have previously shown that it can increase the immunogenicity of different *Neisseria* recombinant proteins in animal immunization studies (23, 26, 29, 30). rNg-ACP proteins were also adsorbed to Al(OH)_3_, the standard adjuvant licensed routinely for human vaccines. Mice were also immunized with the rNg-ACP proteins administered in saline solution alone, as additional controls (14).

The immune response to rNg-ACP proteins was studied initially by the reactivity of individual murine antisera against the homologous protein in an enzyme-linked immunosorbent assay (ELISA) (Table 4). All of the immunizations induced rNg-ACP-specific antibodies, and differences were observed in the geometric mean ELISA titers. The highest geometric mean ELISA titers against the full-length rNg-ACP protein were induced by immunization with protein adsorbed to Al(OH)_3_ (4,230 × 10^3^) and incorporated into liposomes plus MPLA (1,993 × 10^3^). Lower geometric mean antibody levels were induced by protein in ZW 3-14 micelles plus MPLA (164 × 10^3^), saline solution (132 × 10^3^), and, finally, liposomes and ZW 3-14 micelles (14 × 10^3^ to 18 × 10^3^). However, analyses of the nontransformed arithmetic data showed that there were no statistically significant differences between the results measured for the different preparations (*P* > 0.05), as a consequence of the very broad 95% confidence limits observed for many of the animal groups, reflecting differences in murine responses to the protein-adjuvant preparations.

**TABLE 4:** ELISA reactivity of murine antisera raised to full-length and mature rNg-ACP proteins tested against immunizing protein and the densitometry arbitrary units for the corresponding anti-rNg-ACP Western blot bands shown in Fig. 9^*a*^

Immunogen	Adjuvant	Value × 10^3^ of reciprocal geometric mean ELISA titer (95% confidence limits) against immunizing protein	Densitometry arbitrary values for Western blot bands on P9-17 OM (Fig. 9)
Full-length rNg-ACP	Saline	132 (76, 234)	1,835
	Al(OH)_3_	4,230 (232, 77063)	2,438
	Liposomes	18 (4, 84)	672
	Liposomes + MPLA	1,993 (205, 19409)	2,280
	ZW 3-14 micelles	14 (2, 81)	474
	ZW 3-14 micelles + MPLA	164 (60, 449)	812
			
Mature rNg-ACP	Saline	92 (1, 10,880)	99
	Al(OH)_3_	13 (4, 40)	765
	Liposomes	14 (1, 206)	659
	Liposomes + MPLA	239 (1, 94,335)	3,014
	ZW 3-14 micelles	7 (1, 37)	341
	ZW 3-14 micelles + MPLA	16 (5, 46)	428

a All mice were administered three 20-µg doses. Murine antisera were tested in solid-phase ELISA against the respective homologous immunizing recombinant proteins. Data represent the geometric means of the reciprocal ELISA titers of *n* = 5 animals per immunization group, with the 95% confidence limits indicated in parentheses. Serum from sham-immunized animals and normal mouse serum showed no reactivity in ELISA against the recombinant proteins (i.e., the absorbance at λ_450_ nm of antisera tested at a starting dilution of 1/100 was <0.1, i.e., similar to the level seen with no-serum background controls). The intensities of the individual Western blot bands on homologous P9-17 OM for antisera raised to the full-length and mature rNg-ACP proteins shown in Fig. 9 were calculated as arbitrary units of densitometry using ImageJ software.

Immunization with mature rNg-ACP in liposomes plus MPLA also induced the highest geometric mean ELISA titers (239 × 10^3^), followed by protein in saline solution (92 × 10^3^) and then by the remainder of the preparations, i.e., protein with Al(OH)_3_, liposomes, and ZW 3-14 micelles, with and without MPLA (ranging from 7 × 10^3^ to 16 × 10^3^) . Again, there were no statistically significant differences between the results from the different preparations (*P* > 0.05), because of the broad 95% confidence limits (Table 4). Comparisons of the ELISA titers for the mature and full-length protein showed that the two induced similar geometric mean ELISA titers (*P* > 0.05) when administered in saline solution, liposomes, or ZW 3-14 micelles. Although the geometric mean ELISA responses to the full-length protein were higher than those for the mature protein when both were administered in ZW 3-14 micelles plus MPLA, or in liposomes plus MPLA, or, particularly, with Al(OH)_3_ adsorption, they were still not significantly different (*P* > 0.05).

Since the responses to the immunizing protein were statistically similar, we decided to test all of the antisera raised against the rNg-ACP proteins with the different adjuvants by Western blotting and human serum bactericidal activity (hSBA) assays. Western blotting experiments demonstrated that all of the different pooled murine anti-rNg-ACP sera weakly but specifically recognized a single band corresponding to Ng-ACP of *M*_r_ = ∼12,000 in OM of homologous Ng-ACP-expressing strain P9-17 (allele 10) and heterologous Ng-ACP-expressing strain FA1090 (allele 6) (Fig. 9). The intensities of the individual Western blotting bands shown in Fig. 9 for the homologous allele 10 P9-17 full-length and mature rNg-ACP proteins were also calculated as arbitrary units of densitometry using ImageJ software, thereby allowing a qualitative comparison with the mean ELISA titers (Table 4). Checkboards were generated in which the ELISA titers and, similarly, the densitometry values for each individual rNg-ACP-adjuvant preparation were compared, each against the other. In general, there was good concordance between the differences observed in mean ELISA values and the Western blotting densitometry units. For the homologous full-length rNg-ACP protein, there was 80% to 100% concordance between the differences observed in ELISA and in Western blotting for all the different preparations, except for protein administered in saline solution at 60%. As an example, the mean ELISA titer and the Western blot densitometry value for antisera to full-length rNg-ACP-Al(OH)_3_ were higher than for all the other preparations (100% concordance between the assays). This was also true for antisera to mature rNg-ACP-Al(OH)_3,_ but the concordance between the assays was lower for the other preparations, ranging from 20% to 40% for protein in saline solution, Al(OH)_3_, and ZW 3-14 micelles plus MPLA to 60% to 80% for protein in liposomes and ZW 3-14 micelles.

**FIG 9:**
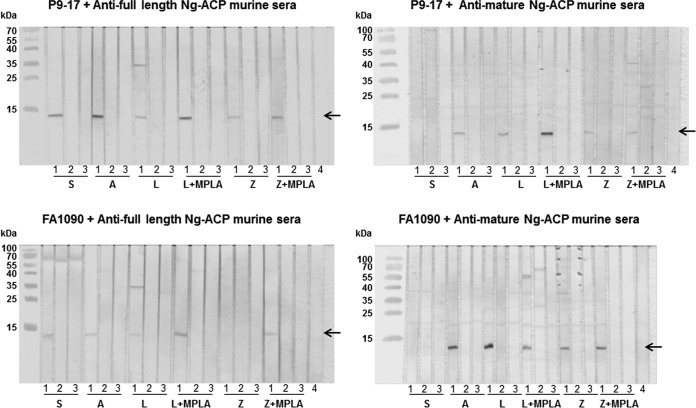
Western blot reactivity of murine antisera to full-length and mature rNg-ACP with outer membranes (OM) of P9-17 and FA1090. Pooled murine antisera to full-length and mature rNg-ACP proteins and control sham immunized sera were reacted (1/100 dilution) against OM preparations of P9-17 and FA1090. In addition, murine antisera were also reacted against the corresponding Δ*ng-acp* strains. Positive reactivity is shown by the arrow denoting a single band of Ng-ACP of *M*_r_ = ∼12,000. In each triplet of strips, “1” denotes the reactivity of antiserum against the wild-type strain; “2” denotes the reactivity of the sham-immunized sera; “3” denotes the reactivity of the antiserum against the Δ*ng-acp* strain; “4” denotes the reactivity of normal mouse serum (NMS). *N* = 3 independent Western blots were done, and representative experiments are shown. S, saline; A, Al(OH)_3_; L, liposomes; L+MPLA, liposomes plus MPLA; Z, ZW 3-14; Z+MPLA, ZW 3-14 plus MPLA.

Specificity in the Western blot assays was confirmed by the absence of reactivity of any of the anti-rNg-ACP sera with the corresponding Δ*ng-acp* mutant strains (Ng-ACP^-^) and the absence of reactivity of sham-immunized sera with Ng-ACP in wild-type bacteria (Fig. 9).

Pooled murine antisera to full-length and mature rNg-ACP proteins were then tested in a human serum bactericidal assay (hSBA) for their ability to kill both homologous P9-17 and heterologous FA1090 strains. Bactericidal antibodies were induced by both proteins when they were delivered with all of the different adjuvant formulations. For antisera to both proteins, reciprocal serum dilutions at which ≥50% killing was observed ranged in general from 64 to 512 against strain P9-17 and from 64 to 256 against strain FA1090 (Table 5). Furthermore, tested against either strain, there were no significant differences (*P* > 0.05) in the bactericidal titers generated by the two proteins when administered with the same adjuvant, except for lower titers recorded by mature protein adsorbed to Al(OH)_3_ (16 to 64) compared to full-length protein (256 to 512). Significantly lower bactericidal titers (*P* < 0.05) were observed for anti-rNg-ACP sera against the corresponding Δ*ng-acp* mutant strains (Ng-ACP^-^), and no significant bactericidal activity was observed for antisera from sham-immunized animals (Table 5). hSBA was also done with murine antisera raised against P9-17 OM and sodium deoxycholate (NaDOC)-extracted–OM preparations, which were shown to react with P9-17 and FA1090 OM in ELISA (Table 6). The bactericidal titers induced by immunization with the OM preparations were 1,024 to 2,048 against strain P9-17 and 256 to 2,048 against strain FA1090, and those induced by the NaDOC-OM preparation were 256 to 1,024 for P9-17 and 256 for FA1090 (Table 6).

**TABLE 5:** Human serum bactericidal activity of murine antisera to full-length and mature rNg-ACP protein delivered with different adjuvants^*a*^

Antigen	Adjuvant	hSBA titer against strain:			
		P9-17 WT	P9-17 Δ*ng-acp*	FA1090 WT	FA1090 Δ*ng-acp*
Full-length rNg-ACP	Saline	256 (256, 1024)	16	128 (64, 128)	4
No antigen	Saline	64	8 (4, 8)	64 (16, 64)	4
					
Mature rNg-ACP	Saline	256	4 (4, 128)	256	64
No antigen	Saline	4	<4	<4	<4
					
Full-length rNg-ACP	Al(OH)_3_	512 (256, 512)	64	256 (256, 512)	64
No antigen	Al(OH)_3_	16	<4	<8	8
					
Mature rNg-ACP	Al(OH)_3_	16–64	16 (4, 64)	16	<4
No antigen	Al(OH)_3_	<4	8	4	<4
					
Full-length rNg-ACP	Liposomes	64	16 (16, 64)	64	4
No antigen	Liposomes	4 (4, 16)	8	≤4	4 (4, 8)
					
Mature rNg-ACP	Liposomes	128–256	4 (4, 64)	64	<4 (<4, 128)
No antigen	Liposomes	4–8	16	4	4
					
Full-length rNg-ACP	Liposomes + MPLA	128 (16, 256)	32	64 (16, 128)	4
No antigen	Liposomes + MPLA	16	4	<4	4
					
Mature rNg-ACP	Liposomes + MPLA	64	16	64	<4
No antigen	Liposomes + MPLA	4	4	<4	<4
					
Full-length rNg-ACP	ZW3-14	64	<4	64	≤4
No antigen	ZW3-14	<4	<4	<4	4
					
Mature rNg-ACP	ZW3-14	64	4	64	<4
No antigen	ZW3-14	4	<4	4	<4
					
Full-length rNg-ACP	ZW3-14 + MPLA	256 (256, 512)	≤4	256 (256, 1024)	16
No antigen	ZW3-14 + MPLA	<4	16	<8	4
					
Mature rNg-ACP	ZW3-14 + MPLA	256 (256, 512)	4	256 (256, 512)	<4
No antigen	ZW3-14 + MPLA	16	4	16	4

a All mice were administered three 20-µg doses. Pooled antisera raised to full-length and mature rNg-ACP using different adjuvants and the corresponding sham-immunized control sera were tested for their ability to induce complement-mediated killing of *N. gonorrhoeae* strain P9-17 (homologous allele 10-encoded Ng-ACP) and FA1090 (heterologous allele 6-encoded Ng-ACP) and their corresponding Δ*ng-acp* variants. The data presented represent the reciprocals of the highest serum dilution at which ≥50% killing was observed. The titers are expressed as the median values from three or more independent experiments and the range of values in parentheses represent the reciprocal hSBA titers for the number of experiments done. No killing was observed with any of the antisera when tested with normal human serum that had been decomplemented by heat inactivation (titers of <4).

**TABLE 6:** ELISA reactivity and human serum bactericidal activity of murine antisera raised to P9-17 OM and NaDOC-OM, tested against homologous P9-17 and heterologous FA1090 strains^*a*^

Immunogen	Dose (µg)/mouse	Adjuvant	Homologous P9-17		Heterologous FA1090	
			Geometric mean ELISA titer × 10^3^ reciprocal (95% confidence limits)	hSBA titer	Geometric mean ELISA titer × 10^3^ reciprocal (95% confidence limits)	hSBA titer
P9-17 OM	3 × 1 µg	Saline	367 (26, 5102)	1,024	47 (23, 97)	1,024
	3 × 10 µg	Saline	581 (40, 8466)	4,096	63 (27, 146)	2,048
	3 × 1 µg	Al(OH)_3_	221 (129, 379)	1,024	33 (15, 71)	256
	3 × 10 µg	Al(OH)_3_	52 (18, 149)	2,048	164 (59, 458)	1,024
P9-17 NaDOC-OM	3 × 1 µg	Saline	3 (0.3, 27)	256–512	1 (0.3, 4)	256
	3 × 10 µg	Saline	112 (30, 421)	256–1,024	10 (2, 39)	256
	3 × 1 µg	Al(OH)_3_	12 (2, 71)	256	18 (4, 74)	256
	3 × 10 µg	Al(OH)_3_	22 (8, 66)	256	108 (25, 466)	256
None	No antigen dose	Saline	No reactivity	16–32	No reactivity	16
	No antigen dose	Al(OH)_3_	No reactivity	16	No reactivity	4

a Murine antisera were tested individually in solid-phase ELISA against the homologous immunizing P9-17 OM and against a heterologous FA1090 OM preparation. Data represent the geometric means of the reciprocal ELISA titers (× 10^3^) of *n* = 5 animals per immunization group, with the 95% confidence limits in parentheses. Sham-immunized animals showed no reactivity in ELISA against the OM preparations (the absorbance at λ_450_ nm of antisera tested at a starting dilution of 1/100 was <0.1; i.e., the level was similar to that measured for the no-serum background controls). Pooled antisera raised to P9-17 OM and NaDOC-OM and corresponding sham-immunized control sera were tested for their ability to induce complement-mediated killing of homologous *N. gonorrhoeae* strain P9-17 (homologous allele 10-encoded Ng-ACP) and FA1090 (heterologous allele 6-encoded Ng-ACP), using an hSBA. The data presented represent the reciprocals of the highest serum dilution at which ≥50% killing was observed. No killing was observed with any of the antisera tested with normal human serum that had been decomplemented by heat inactivation (titers of <4). The titers are expressed as the median values from two or more independent experiments.

To examine whether sialylation had any effect on bactericidal activity, we grew strain P9-17 and FA1090 in the presence of cytidine monophospho-N-acetylneuraminic acid (CMP-NANA) (33, 34) and subsequently tested representative anti-rNg-ACP sera (raised to mature rNg-ACP with ZW 3-14 plus MPLA) and anti-OM sera [raised to 10 µg of P9-17 OM adsorbed to Al(OH)_3_ preparation] in hSBA, alongside testing the antisera against nonsialylated bacteria. In these experiments, the hSBA titers for antisera to both rNg-ACP–ZW 3-14 plus MPLA and to P9-17 OM tested against the nonsialylated strains (Table 5) were reduced to ≤4 against both sialylated strains.

### Antibodies to rNg-ACP inhibit Ng-ACP enzymatic activity.

**FIG 10:**
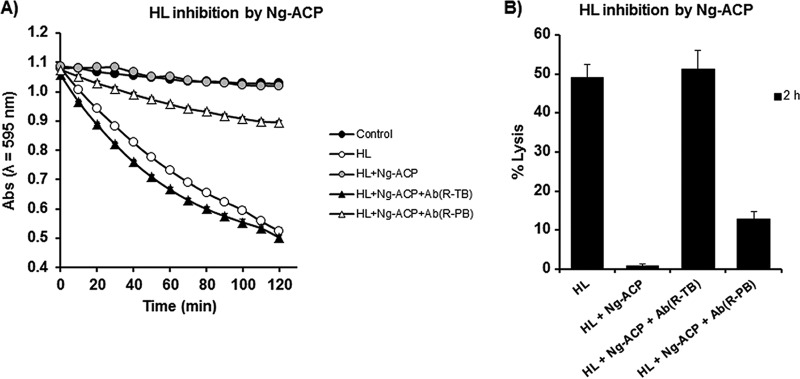
Antibodies to rNg-ACP prevent rNg-ACP from inhibiting HL lytic activity on M. lysodeikticus cells. (A) HL inhibitory activity by rNg-ACP (0.5 µg/ml) was analyzed as a reduction in ODλ_595_ nm (Abs, absorbance) against time for an M. lysodeikticus cell suspension (1 mg/ml) in the presence or absence of decomplemented rabbit anti-rNg-ACP sera (Terminal Bleed [TB]) and sera from the same animal prior to immunization (preimmunization bleed [PB]). The symbols represent the mean absorbance (ODλ_595_ nm) (from *n* = 3 independent experiments), and the error bars represent the corresponding standard errors of the means (SEM). (B) Determination of percentage of M. lysodeikticus cell lysis for each test condition presented in panel A for the time point of 2 h of incubation only. The columns represent mean percent lysis data (from *n* = 3 independent experiments), and the error bars represent the corresponding SEM.

In previous studies (15), we demonstrated that antibodies to rNm-ACP prevented this protein from inhibiting HL *in vitro*. In order to test if the same was true for the gonococcal homologue protein, the ability of decomplemented rabbit species-specific anti-rNg-ACP sera to restore HL lytic activity in the presence of purified rNg-ACP was tested *in vitro*. Antisera to rNg-ACP prevented Ng-ACP from inhibiting HL with ∼100% efficiency, and the specificity was confirmed by the observation of significantly lower levels of inhibition (*P* < 0.05) of rNg-ACP enzymatic activity in the presence of rabbit preimmunization serum (Fig. 10).

## DISCUSSION

The major findings from this study were that (i) *N. gonorhoeae*-ACP was a highly conserved and stably expressed protein; (ii) *N. gonorhoeae*-ACP is structurally conserved to the MliC/PliC family of proteins but lacks conservation MliC/PliC lysozyme recognition motif(s), suggesting a different lysozyme inhibition mode of action; and (iii) rNg-ACP proteins induced antibodies with bactericidal activity toward gonococci expressing the two major Ng-ACP type proteins.

Ng-ACP functions as a human lysozyme inhibitor (15). This is surprising, given that the sequence alignments show that the lysozyme binding motifs are not conserved (Fig. 3) and that the structural analyses and superposition with lysozyme complexes of PliC/MliC proteins reveal clashes (Fig. 2E). This is also apparent from structural superposition of Ng-ACP with PliC (Fig. 2D), which reveals substantial differences in the putative interface region. Ng-ACP is very similar to Nm-ACP (Fig. 2C), and a docking exercise was previously used on Nm-ACP to understand whether lysozyme binding through this interface would still be possible: that study showed that the lysozyme, if it was bound in this position, would have to be significantly shifted away from Nm-ACP, compared to the Ba-PliC-lysozyme interaction (15). The study noted that loop flexibility in the regions that clash was unaccounted for using the approach taken (15). We have now used a conservation mapping of ACP sequences available from 10 *Neisseria* species, revealing that the interface is not entirely conserved. In particular, conservation is lacking in the β6 region that forms an important part of the interface. Together with the clashes seen around helix α1 of Ng-ACP, not present in PliC (Fig. 2D), interaction using this interface seems unlikely. However, the analysis also reveals conservation in the loop 4 region (Fig. 4), which is involved in lysozyme recognition, and hence interaction might occur through this region using a different binding mode; this would explain the difference in sequence length in this region between Ng-ACP and PliC proteins. The conservation mapping also reveals a conserved region at the C-terminal end of Ng-ACP, involving residues found in the β7 to β8 region (Fig. 4). It is perhaps significant that β8 is stabilized by the conserved cysteine bridge to β1 (Fig. 2A). In the absence of a *Neisseria* ACP-lysozyme complex structure, these two regions should be targeted for mutagenesis to reveal their significance in lysozyme inhibition.

The similarity to the MliC/PliC protein family suggests that Ng-ACP may probably be located in the periplasm or inner leaflet of the OM. We have reported that anti-rNm-ACP antibodies reacted with OM from N. meningitidis strain MC58 in ELISA, bound specifically to whole meningococci as judged by flow cytometry measurements, and induced bactericidal antibodies (14, 15), all of which suggested surface exposure of this protein. In contrast, we were unable to observe binding of anti-rNg-ACP antibodies to gonococcal OM in ELISA or whole gonococci by flow cytometry. One possible explanation could be the relative differences in ACP expression, with lower levels observed for P9-17 OM than for MC58 OM. However, specific reactivity was observed in Western blots of anti-rNg-ACP sera with gonococcal OM and our recent studies suggest that Ng-ACP appears to be released extracellularly to an extent (35). These observations, along with the specifically induced bactericidal activity, suggest surface accessibility of Ng-ACP to antibody binding.

Antisera to rNg-ACP proteins were bactericidal not only for the homologous P9-17 reference strain (allele 10) but also for the heterologous FA1090 reference strain (allele 6) tested. Thus, immunization with a single allelic protein could potentially provide coverage of ≥96% of the gonococcal isolates in the https://pubmlst.org/neisseria/ database with defined alleles. However, the cross-protective bactericidal activity of anti-rNg-ACP sera would need to be examined with a larger number of allele 10- and allele 6-expressing strains to test this hypothesis. Although it is difficult to compare results from published studies, due to the use of different gonococcal strains and the absence of a standardized hSBA, the rNg-ACP-induced bactericidal titers, ranging from 64 to 512, were similar to the bactericidal immune responses induced by other candidate gonococcal antigens that have been reported to be stably expressed and highly conserved. Recently, recombinant proteins were prepared of candidate antigens that were identified by proteomics and bioinformatics analyses, and several induced murine antisera with bactericidal activity, e.g., α-BamA (NGO1801), NGO2054, MetQ (NGO2139), TamA (NGO1956), and LptD (NGO1715), with titers ranging from 16 to 512 against different strains (36, 37). Other examples include proteins involved in colonization and invasion, e.g., PilQ_406-770_ (titers of 400 to 800) (38); nutrient acquisition, e.g., transferrin-binding proteins (titers of 50 to 100) (39); and immune evasion, e.g., porin peptides (titers of ∼320) (40), NspA (titers of ∼100 to 1,000) (41), and LOS 2C7 mimotopes (with percentages of killings calculated per microgram of IgG) (42), among others (5, 7, 43).

In the current study, and, indeed, in the studies performed with the other candidate antigens described above, hSBAs were done with bacteria grown without CMP-NANA. N. gonorrhoeae has the ability to scavenge CMP-NANA from the urogenital tract and to use it to sialylate LOS, which renders the organism resistant to killing by complement, which is present in genital secretions and normal human serum (44). Freshly isolated gonococci are generally serum resistant; this property is rapidly lost upon laboratory subculture (45) but can be restored by treatment with exogenous CMP-NANA *in vitro.* The sialylated nature of the gonococcal strains used in hSBAs may have a profound influence on whether antibodies to candidate vaccine antigens can kill bacteria via complement activation. It has been well documented that LOS sialylation prevents complement-dependent killing by immune sera generated in response to different OM antigens (33, 34, 46). More recently, Ram and colleagues demonstrated how increasing amounts of CMP-NANA sialylation reduced the bactericidal activity of the 2C7 monoclonal antibody (47). Concentrations of >3 µg/ml of CMP-NANA led to >50% survival of gonococci in response to the effects of the bactericidal antibody.

In our study, we found, not unexpectedly, that sialylation of gonococci inhibited the bactericidal effects of representative antisera produced in response to both rNg-ACP and OM preparations. Whether abrogation of bactericidal activity against sialylated bacteria precludes the inclusion of rNg-ACP and other candidate OM antigens in new gonococcal vaccines is a matter of conjecture. Generation of antibodies with bactericidal activity against unsialylated gonococci present during infection still has merit. LOS sialylation of gonococci *in vivo* probably covers excess sialylation, which may block invasion of gonococci into mucosal epithelial cells but protects the organism from natural host immunity, to loss of sialic acid modification, which conversely makes the gonococcus more invasive but more susceptible to eradication by host immune mechanisms (48). Furthermore, Schneider and colleagues (49) used a human male challenge model to demonstrate that gonococci with sialylated LOS were less infective than gonococci with nonsialylated LOS. More recently, Ketterer and colleagues (50) showed that cervical secretions obtained from women infected with gonorrhoea contained levels of sialidases that could remove sialic acid from sialylated LOS, thereby rendering the gonococci potentially susceptible to bactericidal antibodies that normally may have no activity against sialylated bacteria. Notwithstanding the fact that LOS sialylation occurs *in vivo* to some extent, complement-mediated killing may still be induced by targeting the immune response to surface-exposed epitopes on OM antigens, which show the least susceptibility to the potential inhibitory effect of LOS sialylation (34).

In our study, ELISA demonstrated differences in the geometric mean levels of antibodies induced by rNg-ACP proteins with the different adjuvants, although the data were not statistically significant due to large confidence limits within each individual group of animals. Regardless, all of these antisera were able to recognize Ng-ACP in OM preparations. The ability of all the protein-adjuvant preparations to induce bactericidal antibodies suggested that this functional activity was independent of the total mean quantitative antibody amount. Qualitative differences probably exist between the antibodies produced with the different adjuvants, and antibodies of the correct isotype and of high avidity are likely to contribute to bactericidal activity. We did not measure the antibody isotype or the avidity of our anti-rNg-ACP sera, as the wide confidence limits in comparisons between the data from the animals in each immunization group might not have provided statistically significant differences. In mice, the IgG2a isotype is the most efficient activator of complement (51–53), but there is limited information in the literature on the isotypes of murine antibodies to gonococcal antigens that are preferred for complement fixation and hSBA. Price and colleagues showed that IgG2a was the main isotype of antibodies to gonococcal transferrin-binding protein associated with bactericidal activity (54). In addition, we have observed in previous studies that addition of adjuvants can increase the levels of murine IgG2 antibody isotypes relative to IgG1 isotypes, e.g., MPLA introduced into meningococcal rPorB liposomes and r-PorB micelles (24) and MPLA introduced into meningococcal rOpc liposomes (23). However, in humans, immunization should favor IgG1 and IgG3 isotypes, which are the most efficient activators of complement, whereas IgG2a is effective only at high epitope densities (55, 56).

Several transcriptome analyses have reported that *ng-acp* gene expression was upregulated in response to hydrogen peroxide (57) and was induced in gonococci grown anaerobically and under iron-depleted conditions (58) and that *ng-acp* transcriptional regulation might be Fur dependent (59, 60). However, none of those studies examined levels of protein expression. In our study, Ng-ACP protein was expressed by all 50 gonococcal isolates in the CDCP/FDA AR Isolate Bank, with some minor variability in the relative levels of expression. No correlation was found between the levels of Ng-ACP expression and the antimicrobial resistance patterns described for the isolates in the CDCP/FDA bank.

Recently, we showed that Ng-ACP is important for gonococcal survival challenge not only by purified HL but also by human tears, saliva, and adherent interleukin-8 (IL-8)-treated primary neutrophils, which are heavily recruited to sites of gonococcal infection and constitute the major source of HL that this pathogen might encounter (35). Furthermore, Δ*acp* gonococci and Δ*acp* meningococci are significantly more sensitive to HL than the corresponding wild-type strains (15). In the current study, we demonstrated that antibodies to Ng-ACP could also prevent the protein from inhibiting HL lysis of Micrococcus lysodeikticus *in vitro*. Thus, in summary, Ng-ACP is a stable, highly conserved protein that functions principally as a lysozyme inhibitor (15). Ng-ACP is a potential dual target for tackling gonococcal infections, as inclusion of this protein within a multicomponent vaccine could induce antibodies that are bactericidal and could also prevent the gonococcus from inhibiting the lytic activity of a mucosal innate defense molecule.

## MATERIALS AND METHODS

### Bacteria and growth conditions.

Neisseria gonorrhoeae strain P9-17, a 1B-26 serovar isolate (ND: P1.18-10,43: F1-26: ST-1926), was originally isolated from a patient with gonococcal prostatitis (45). N. gonorrhoeae strain FA1090 (ATCC 700825) was purchased from the American Type Culture Collection. The panel of 50 N. gonorrhoeae isolates assembled by the Centers for Disease Control and Prevention (CDCP) in collaboration with the Food and Drug Administration (FDA) was also obtained (Table 3). These isolates represent a diversity of levels of antimicrobial susceptibility to drugs that are used to treat infections, and their genomes have been sequenced (https://www.cdc.gov/drugresistance/resistance-bank/currently-available.html).

Gonococci were grown on supplemented gonococcal (GC)-agar plates (61) incubated at 37°C in an atmosphere containing 5% (vol/vol) CO_2_. Escherichia coli strains DH5α (cloning) and BL21(DE3)pLysS (protein expression) were grown at 37°C on Luria Bertani (LB) agar and in LB broth. Outer membranes (OM) of P9-17 and FA1090 bacteria were prepared and extracted with sodium deoxycholate (NaDOC) as described previously (62, 63).

### Cloning and expression of *ng-acp* genes in Escherichia coli.

Genomic DNA of N. gonorrhoeae P9-17 was extracted by alkaline lysis, as described previously (26), and used as the PCR template. The method for gene cloning into the pRSETA system (Invitrogen) was described previously (26). The gene sequence of P9-17 *ng-acp* (NEIS2075, https://pubmlst.org/neisseria/, 372 bp) was amplified by PCR using the forward primer seq2095F (5′-GGCTATCTCGAGATGAAACTTCTGACCACTGC-3′) and the reverse primer seq2095R (5′-GGCTATAAGCTTCTATTAACGTGGGGAACAGTCTT-3′). The CTCGAG and AAGCTT sequences represent the restriction sites for XhoI and HindIII enzymes, respectively.

The method for cloning of *ng-acp* into the pET22b vector (Novagen) has been described previously (15). Recombinant plasmids (pRSETA-*ng-acp*, pET22b-*ng-acp*) carrying the P9-17 *ng-acp* genes were transformed into E. coli DH5α cells for plasmid amplification and subsequently into E. coli BL21(DE3)pLysS for protein expression. For optimal protein expression, isopropyl-β-d-thiogalactopyranoside (IPTG) was added to E. coli BL21(DE3)pLysS cultures in LB broth to reach a final concentration of 1 mM, followed by bacterial growth for 4 h.

### Purification of recombinant rNg-ACP proteins.

rNg-ACP proteins were purified using nickel-nitrilotriacetic acid (Ni-NTA) metal-affinity chromatography under denaturing and native conditions (QIAexpressionist system manual; Qiagen, United Kingdom). Pilot expression trials of rNg-ACP expressed from E. coli BL21(DE3)pLysS-pRSETA-*ng-acp* demonstrated that the recombinant protein was insoluble and required suspension in 100 mM NaH_2_PO_4_–10 mM Tris-HCl (pH 8.0) buffer containing 6 M guanidinium chloride (GuHCl) (14). Protein bound to Ni-NTA resin was eluted using 100 mM NaH_2_PO_4_–10 mM Tris buffer containing 6 M GuHCl (pH 4.5) and precipitated by adding trichloroacetic acid (TCA; BDH, United Kingdom) to reach a 5% (wt/vol) final concentration, and the precipitate was solubilized in phosphate-buffered saline (PBS) containing 0.5% (wt/vol) SDS. In contrast, rNg-ACP expressed from E. coli BL21(DE3)pLysS-pET22b-*ng-acp* was soluble and was purified using native conditions, as described previously (15), with bound protein eluted using 50 mM NaH_2_PO_4_–300 mM NaCl–250 mM imidazole buffer (pH 8.0) and subsequently dialyzed against PBS (pH 7.4) for 48 h. Samples obtained during the purification procedures were analyzed using 14% (wt/vol) SDS-PAGE as described previously (22), and protein concentrations were determined using the bicinchoninic acid (BCA) assay (Pierce, Thermo-Scientific, United Kingdom).

### Crystal structure of rNg-ACP.

To produce rNg-ACP for X-ray crystallography, E. coli BL21(DE3) pLysS-pET22b-*ng-acp* was grown in 6 to 8 liters of LB broth. Cultures were centrifuged (9,000 × *g* for 30 min at 4°C on an Avanti J-30I high-performance centrifuge; Beckman Coulter), and the cell pellet was suspended in 125 ml of lysis buffer (50 mM Tris HCl buffer [pH 8.5] containing 300 mM NaCl and 10% [vol/vol] glycerol) and subjected to probe sonication on ice (Misonix Sonicator Xl2020 ultrasonic processor) (10,000 amplitude for 10 s on and 30 s off over 5 min) followed by clarification by ultracentrifugation (Beckman Coulter XPN) (1,000 × *g* for 45 min at 4°C). The clarified supernatant was subjected to filter sterilization (using a 0.22-μm-pore-size filter) and purified using a Ni-His trap HP column (GE Healthcare) (1 ml) on an Akta Prime liquid chromatography system (GE Healthcare), equilibrated with lysis buffer. Elution buffer contained 50 mM Tris-HCl buffer (pH 8.5), 300 mM NaCl, and 10% (vol/vol) glycerol, and two wash steps were performed with 20 mM and 40 mM imidazole before elution of the protein was performed with 300 mM imidazole.

Elution fractions were combined and concentrated using a Vivaspin-20 5,000-molecular-weight-cutoff (MWCO) polyethersulfone (PES) concentrator (Sartorius). Subsequent gel filtration used a high-load 16/600 Superdex 75-pg size exclusion column (GE Healthcare) on an Akta Prime chromatography system (GE Healthcare). Fractions containing Ng-ACP as judged by SDS-PAGE (single band with *M*_r_ of ∼12,000) were combined and concentrated (Vivaspin-20 5,000-MWCO PES concentrator) to a concentration of 50 mg/ml or higher as determined using a NanDrop 2000 spectrophotometer (Thermo Scientific). Purification resulted in a single band of rNg-ACP (*M*_r_ = ∼12,000) as analyzed by SDS-PAGE (Fig. 1B and C).

Crystallization used an Art Robbins Gryphon nanodrop dispenser (Art Robbins Instruments) with a 96-well Intelli-Plate (Art Robbins Instruments) for sitting-drop vapour diffusion and Pact *premier* pH, anion, and cation crystallization kits (Molecular Dimensions, United Kingdom) (64), followed by manual optimization. Optimal crystallization was carried out at a temperature of 4°C with 0.1 M potassium thiocyanate (KSCN) and 30% (wt/vol) polyethylene glycol methyl ether 2000 (PEG2000MME).

Diffraction data from rNg-ACP crystals were collected at the beamline ID23-1 European Synchrotron Radiation Facility, Grenoble, France. Data were integrated with XDS (65) and scaled with Aimless (66). Molecular replacement was carried out with Molrep (67), followed by iterative model building and refinement using Coot (68) and refmac5 (69). All other data manipulations were carried out with programs from the CCP4 suite (70). Models were validated using the ePDB validation server (71).

In addition, the sequence of Ng-ACP was used as the input in the NIH BLASTP tool (72) to identify homologues in the database of nonredundant protein sequences. From the output, 10 sequences encoding adhesin proteins from different *Neisseria* species were selected and aligned using ClustalW (73). The alignment and the Ng-ACP structure were used as the input in ConSurf (18), and the conservation mapping was displayed with Chimera (74).

### Immunization of mice with mature and full-length rNg-ACP proteins and adjuvants.

BALB/c mice (H-2^d^ haplotype) were bred within the animal facilities of the university under standard conditions of temperature and humidity with a 12-h lighting cycle and with food and water available *ad libitum.* Groups of five BALB/c mice of approximate equal sizes and weights (6 to 7 weeks of age) were immunized intraperitoneally with purified mature or full-length rNg-ACP protein prepared separately with the following adjuvant and delivery vehicles.

**(i) Saline solution.** A solution of 200 µg/ml of full-length or mature rNg-ACP was made in sterile saline solution (0.9% [wt/vol] NaCl), and each mouse (*n* = 5 mice) was immunized with a 100-µl volume containing 20 µg of protein. Control mice (*n* = 5) were immunized with saline solution alone (100 µl).

**(ii) Adsorption to Al(OH)_3_.** A volume of 0.35 ml of a solution of 400 µg/ml of full-length or mature rNg-ACP in sterile saline solution was mixed with 0.35 ml of aluminum hydroxide [Al(OH)_3_; Alhydrogel, Superfos] suspension. The mixture was placed on a rotary mixer overnight at 4°C to allow protein adsorption. A control preparation without protein was produced similarly. Each mouse (*n* = 5 mice) was immunized with 100 µl of Al(OH)_3_-adsorbed protein (containing 20 µg protein) and control mice (*n* = 5) with adjuvant alone.

**(iii) Liposomes.** Liposomes were prepared following a protocol described previously (14, 21). A lipid shell was prepared in 70% (vol/vol) nitric acid round-bottomed flasks containing 20 mg of a 7:2 molar ratio of l-α-phosphatidylcholine/cholesterol. l-α-Phosphatidylcholine (87.5 μl of a 100 mg/ml stock) was added directly to a round-bottomed flask followed by cholesterol (125 μl of a 10 mg/ml cholesterol solution dissolved in chloroform), and the volume was adjusted to 3 ml with chloroform. Dried lipid shell was produced by evaporating the solvent at 25°C using a rotary evaporator (Buchi). A solution of 50 mg of octyl-β-glucopyranoside was prepared in 10 mM HEPES (pH 7.2) buffer and incubated at room temperature for 3 h. Full-length rNg-ACP or mature rNg-ACP was suspended in PBS–0.5% (wt/vol) SDS or in PBS alone, respectively, to reach a final concentration of 500 μg/ml. A control liposome was made without protein added. The dried lipid shell was suspended with constant manual agitation in the protein-octyl-β-glucopyranoside solution or in the control octyl-β-glucopyranoside solution, and the mixtures were left at room temperature for 1 h. The liposome solutions were dialyzed against PBS at 4°C for 72 h with two changes of buffer daily (to remove detergents) and then transferred to Bijoux tubes, and small unilamellar liposomes were generated by sonication using a MSE Soniprep 150 probe sonicator (15 to 20 times for 30 s each time on ice; amplitude of 10 to 15 μm). Final volumes of liposome preparations were measured, and the preparations were stored at −20°C. Each mouse (*n* = 5) was immunized with liposomes containing 20 μg protein and control mice (*n* = 5) with empty liposomes alone.

**(iv) Liposomes plus monophosphoryl lipid A (MPLA).** The adjuvant MPLA (500 μg of S. enterica serotype Minnesota; Sigma-Aldrich) was dissolved in chloroform and added to the l-α-phosphatidylcholine–cholesterol mixture, and a dried lipid shell was prepared as described above. Protein-containing and control liposomes with MPLA were prepared as described above. Each mouse (*n* = 5) was immunized with liposomes containing 20 μg protein and 20 μg MPLA and control mice (*n* = 5) with liposomes containing 20 μg MPLA alone.

**(v) Zwitterion (ZW) detergent 3-14.** A stock solution of 80 mg of Zwitterion detergent ZW 3-14 (Calbiochem) was prepared in 1 ml of sterile saline solution. Solutions of full-length or mature rNg-ACP were prepared in saline solution to give a final concentration of 500 μg/ml. The detergent mixture was prepared by adding 100 μl of ZW 3-14 stock solution (to give a final concentration of 8 mg/ml) to the volume containing 500 μg/ml of full-length or mature rNg-ACP, and the final volume was adjusted to 1 ml with sterile saline solution. This mixture was kept at room temperature overnight to allow micelle formation, and the stock was diluted with sterile saline solution to reach a final concentration of 200 μg/ml. Each mouse (*n* = 5) was immunized with 100 µl of ZW 3-14–protein solution containing 20 µg protein and control mice (*n* = 5) with ZW 3-14 solution alone, prepared without the addition of protein. Unused stock solution was stored in aliquots at −20°C for subsequent immunizations.

**(vi) Zwitterion (ZW) detergent 3-14 with monophophoryl lipid A (MPLA).** MPLA was suspended in saline solution to reach a final concentration of 1 mg/ml. A 50-µg volume of MPLA and a 50-μg volume of full-length or mature rNg-ACP were added to a solution of 8 mg/ml ZW 3-14, and the volume was adjusted to 1 ml with saline solution. The solution was kept at room temperature overnight. Each mouse (*n* = 5) was immunized with 20 μg of full-length or mature rNg-ACP in ZW 3-14–20-μg MPLA, and control mice (*n* = 5) were immunized with the same mixture without protein.

Mice were immunized on days 0, 14, and 28. One group (*n* = 5) was kept for use as a source of normal mouse serum (NMS). All mice were terminally bled by cardiac puncture under anesthesia on day 42.

### Immunization of mice with OM preparations.

Groups of five BALB/c mice were immunized with P9-17 OM and NaDOC-OM preparations (doses of 1 µg/mouse and 10 µg/mouse) in saline solution and adsorbed to Al(OH)_3_ using the same schedule as described above for the recombinant proteins. In order to limit the potential toxicity from the increasing amounts of LOS in the native OM preparations, higher doses of OM were avoided for immunization. Groups of five mice were also subjected to sham immunization (no protein or OM or NaDOC-OM), and one group was kept for use as a source of normal mouse serum (NMS). Mice were terminally bled by cardiac puncture under anesthesia on day 42.

### Immunization of rabbits with rNg-ACP.

Rabbits (*n* = 2) were immunized subcutaneously with full-length rNg-ACP using the services of David Biotechnologie GmbH, Regensburg, Germany. Rabbits were immunized with rNg-ACP (100 µg per dose per rabbit) emulsified in Freund’s complete adjuvant for the primary injection (day 0) and Freund’s incomplete adjuvant for a subsequent four injections performed at ∼14-day intervals, with terminal bleeding on day 63. Postimmune rabbit antisera to full-length rNg-ACP showed high reactivity in ELISA against homologous protein (mean reciprocal ELISA endpoint titer of ∼9 × 10^6^) and mature protein (mean reciprocal ELISA endpoint titer of ∼1 × 10^6^) and reacted weakly in ELISA with OM from P9-17 (mean reciprocal ELISA endpoint titer of ∼7,000) and FA1090 (mean reciprocal ELISA endpoint titer of ∼9,000).

All murine and rabbit sera were stored at −20°C until required and were decomplemented by heating at 56°C in a water bath for 30 min before use.

### Animal ethics statement.

This study complied with the animal experimentation guidelines of the Home Office (HO), with approval granted under the Animals Scientific Procedures Act, 1986, with HO project license number PPL 30/3126. The study was approved by the Animal Welfare and Ethics Review Board at the institution with which most of us are affiliated (University of Southampton; no number assigned). Animal health and welfare were assessed daily by qualified animal technicians, and no animals suffered significant adverse effects. Davids Biotechnologie GmbH has a permit from the Veterinäramt Regensburg for housing specific-pathogen free, healthy rabbits according to §11 TierSchG (Az31.4.4/ScP1). The company is registered for immunization of animals under Aketenzeichen AZ 2532.44/14 by the approving authority, Umweltamt Regensburg/Veterinärwesen. All immunizations were done in accordance with National Institutes of Health standards for animal welfare (NIH animal welfare number A5646-01).

### Characterization of biological and functional properties of antibodies to rNg-ACP.

**(i) ELISA.** Individual murine antisera were reacted in ELISA against both rNg-ACP proteins and P9-17 and FA1090 OM, as described previously (22). Absorbance was measured at λ_450_ nm after 10 min of incubation with enzyme substrate, and the ELISA titer, extrapolated from the linear portion of the serum titration curve, was taken as the reciprocal of the dilution which gave an increase in absorbance of 0.1 U after 10 min. One-way analysis of variance (ANOVA) with Dunnett’s multiple comparison test was used on nontransformed arithmetic data to compare mean values for ELISA data, with *P* values of <0.05 considered significant.

**(ii) Western immunoblotting.** Samples containing rNg-ACP proteins, OM, and/or whole-cell lysate preparations were separated on SDS-PAGE and then transferred to nitrocellulose by semidry blotting. After incubation with murine or rabbit sera, immunological reactivity was detected by using anti-mouse/rabbit immunoglobulin-alkaline phosphatase conjugate (Bio-Rad, United Kingdom) as described previously (22).

**(iii) Examining Ng-ACP expression in the CDCP/FDA panel of gonococcal isolates by Western blotting.** Individual bacterial lysates (*n* = 50 for the CDCP/FDA panel and also P9-17 and FA1090) were prepared in phosphate-buffered saline (PBS) (pH 7.4), subjected to heat inactivation for 1 h at 56°C in a water bath, and briefly sonicated with a probe (MSE Soniprep). Protein concentrations were estimated by BCA assay (Pierce). Linear gels were prepared (14% [vol/vol] acrylamide), and 50 µg of bacterial protein was loaded in triplicate wells. Electrophoresis was run at 200 V for 50 to 60 min, and proteins were transferred to nitrocellulose at 10 V for 30 min. For antibody blotting reactions, nitrocellulose was reacted with rabbit anti-rNm-ACP serum (1/20 dilution), which cross-reacts with Ng-ACP protein (15), and with monoclonal antibody Ab-anti-LPDA(Nm) (Abcam catalog no. ab80913) (1/3,000 dilution), which recognizes the conserved OM protein dihydrolipoyl dehydrogenase (LPDA). Binding was detected using donkey anti-rabbit lgG-alkaline phosphatase and goat anti-mouse lgG (H+L)-alkaline phosphatase conjugates (1/3,000 dilution).

Nitrocellulose gel images were scanned, and band densitometries were calculated in pixels with the ImageJ program. The ratios of Ng-ACP/LPDA densitometry values were determined in triplicate with standard deviations. Statistical differences in ratio values were analyzed for each isolate against P9-17 as a reference using a two-sample *t* test (assuming unequal variances). For ratios lower than those seen with P9-17, *P* values of <0.05 denoted significance; for ratios higher than the P9-17 ratios, *P* values of <0.05 also denoted significance; for ratios similar to the P9-17 ratios, *P* values of ≥0.05 denoted no significant differences.

**(iv) Comparing ACP expression levels in Neisseria meningitidis and Neisseria gonorrhoeae OM preparations.** OM (30 µg/well) from N. meningitidis strain MC58 and OM from N. gonorrhoeae strain P9-17 were separated by SDS-PAGE and then transferred to nitrocellulose by semidry blotting. Blots were reacted with rabbit antiserum raised to rNg-ACP (rabbit 1 and rabbit 2, at 1/20 dilution) or with the rabbit anti-rNm-ACP serum (1/20 dilution), which cross-reacts with Ng-ACP protein, and, concomitantly, with monoclonal antibody Ab-anti-LPDA(Nm) (Abcam catalog no. ab80913) (1/3,000 dilution), which recognizes the conserved OM protein LPDA. Binding was detected using donkey anti-rabbit lgG-alkaline phosphatase and goat anti-mouse lgG (H + L)-alkaline phosphatase conjugates (1/3,000 dilution). The ratios of ACP/LPDA densitometry values were determined as described above, and statistical differences in ratio values between OM preparations for each rabbit antiserum tested were analyzed using a two-sample *t* test (assuming equal variances), with *P* values of <0.05 considered significant.

**(v) Flow cytometry.** Binding of murine and rabbit antibodies to P9-17 and FA1090 was examined by flow cytometry, as described previously (14). An overnight culture of bacteria grown for ≤16 h was collected by centrifugation, and bacteria were washed twice with sterile PBS containing 1% (wt/vol) bovine serum albumin (BSA) and suspended to ∼2 × 10^8^ CFU/ml. Next, bacteria (l ml) were centrifuged (2,200 × *g* for 3 min), suspended in 200 μl of rabbit sera or pooled murine sera (with various dilutions of both species serum tested, from 1/10 to 1/400), and incubated at 37°C for 30 min. After washing with PBS was performed, bacteria were incubated with 100 μl of fluorescein isothiocyanate (FITC)-conjugated goat anti-rabbit or rabbit anti-mouse IgG (Dako, United Kingdom) (1/50 dilution) at room temperature for 30 min. Bacteria were fixed with a 0.4% (wt/vol) paraformaldehyde solution at room temperature for 30 min. Samples were analyzed on a FACSAria flow cytometer (BD Biosciences, USA).

**(vi) Determination of the bactericidal activity of anti-rNg-ACP sera for N. gonorrhoeae using a human serum complement bactericidal assay (hSBA).** The hSBA was based on the protocol described by McQuillen et al. (75) with modifications (30, 40). Gonococci were grown overnight on GC agar plates for ≤16 h, and assays were done in sterile 96-well microtiter plates with lids (Greiner Bio-One), with wells containing a mixture of 25 µl of bacteria (∼1,000 CFU) in Dulbecco’s modification of PBS (PBSB), 17 µl of normal human serum (NHS) as the exogenous complement source, and 10 to 25 µl of serial dilutions of pooled test serum, and were adjusted to a final volume of 100 µl with PBSB containing 1% (vol/vol) decomplemented fetal calf serum (dFCS). NHS from a single source (a laboratory staff member with no history of gonococcal or meningococcal infection and with written consent provided) was used for all of the hSBA experiments and prescreened for its ability to support complement-mediated killing of gonococcal strain P9, using sera raised to OM as a reference antiserum of known bactericidal activity. The collection of blood was approved by and done in accordance with the ethical standards of the National Research Ethics Service South Central—Hampshire A Committee (13/SC/0416). Control wells contained no serum, or serum from sham-immunized animals, but did contain decomplemented NHS prepared by heat inactivation in a water bath at 56°C for 30 min, to prove that bactericidal activity was due to a complement-mediated mechanism and not to other factors present in the mouse sera. To confirm that the bactericidal assay was working, each assay included wells containing anti-P9 OM serum of known bactericidal activity, e.g., at the 50% endpoint serum dilution, as a positive control. Plates were incubated for 1 h at 37°C in a humidified atmosphere with 5% (vol/vol) CO_2_. Aliquots of 15 µl were plated in triplicate on GC agar plates and colonies counted at 24 to 48 h later (ProtoCOL; Synoptics Ltd., Cambridge, United Kingdom). Human serum complement-dependent bactericidal activity was determined from the numbers of bacteria surviving in the presence of serum and complement compared to the numbers surviving with complement but without test serum. Sera that showed (>50%) bactericidal activity in two or more dilutions were considered positive. The 50% hSBA titers for each serum pool were determined from a minimum of *n* = 3 independent experiments and are presented as the median values with the range observed within the experiments.

Representative hSBA experiments were done similarly, with gonococci grown in the presence of cytidine-5′-monophospho-N-acetylneuraminic acid (CMP-NANA) (33, 34). Briefly, 1 ml of a 1 mg/ml solution of CMP-NANA–sodium salt (Sigma) was sterilized by filtration, spread onto a surface of a 20-ml GC agar plate, and allowed to diffuse into the agar to give a final concentration of 50 µg/ml (33, 34). Single colonies grown on plates without CMP-NANA for 16 h at 37°C in an atmosphere of 5% (vol/vol) CO_2_ were grown as a lawn on the CMP-NANA plates for a further 16 h. Sialylated bacteria were then harvested and immediately used in hSBAs as described above.

**(vii) Human neutrophil lysozyme (HL) inhibition assays.** A lysozyme inhibition assay was done using freeze-dried M. lysodeikticus cells (ATCC 4698; Sigma-Aldrich) suspended at 1 mg/ml in 10 mM potassium phosphate buffer (PPB) (pH 7.0) supplemented with a protease inhibition cocktail (Roche), as described previously for Nm-ACP (15). To test the ability of antiserum to rNg-ACP to block Ng-ACP function as a HL inhibitor, a 1/16 dilution of decomplemented rabbit anti-rNg-ACP sera was added to the reaction mixture and HL activity was measured every 5 min for 2 h. The corresponding preimmunization rabbit serum was included as the negative control.

### Sequencing the *ng-acp* gene of N. gonorrhoeae strain P9-17.

The *ng-acp* gene of N. gonorrhoeae strain P9-17 was sequenced commercially (Geneservice, Oxford, United Kingdom) using the primer Seq2095 (5′-CGGGATACGCCGACATTAGA-3′). Whole-genome sequencing of genomic DNA extracted from P9-17 was done on an Illumina HiSeq 2500 system at the Department of Zoology Sequencing Facility, University of Oxford. The identification number for P9-17 in https://pubmlst.org/neisseria/ is 36675.

### Constructing *ng-acp* knockout mutants.

Mutagenesis was achieved by heterologous allelic exchange, following a protocol described previously (15). Initially, two pairs of primers were designed to amplify the fragments up- and downstream of the *ng-acp* gene and to include the restriction enzyme XbaI sequence (5′-CTAGA-3′) for assembly of the construct fragments. Using genomic DNA from N. gonorrhoeae strain P9-17 as the template, the upstream fragment (F1) of the *ng-acp* gene was PCR amplified (Phusion High-Fidelity PCR Master Mix, BioLabs) using a forward primer (sequence 5′-TAGACTTCTGGGGCAAGGTC-3′) and reverse primer (sequence 5′- GGCTATTCTAGATTTTATTCCTTTGGATAGATG-3′), with the conditions consisting of initial denaturation (98°C, 30 s); 30 cycles of denaturation (98°C, 10 s), annealing (55°C, 30 s), and extension (72°C, 14 s); and a final extension step (72°C, 5 min). The downstream fragment (F2) was amplified with forward primer 5′-GGCTATTCTAGATCAGGCAACAAAAAACAGCG-3′ and reverse primer 5′-GGTACGGAGATTGTCGCCC-3′ using similar PCR conditions, except for the annealing step (63°C, 30 s). A kanamycin (Kan) antibiotic resistance cassette (from APCYC177; New England BioLabs) was amplified for insertion between the up- and downstream fragments of the *ng-acp* gene. A *Neisseria* DNA uptake sequence (DUS) (76) was also inserted to enable DNA uptake. The Kan cassette was amplified using forward primer 5′-GGTTCTAGATTCAGACGGCGTGATCTGATCCTTCAACTC-3′ and reverse primer 5′- GGTTCTAGATTAGAAAAACTCATCGAGCATC-3′, with PCR conditions of initial denaturation (98°C, 30 s); 30 cycles of denaturation (98°C, 10 s), annealing (54°C, 30 s), and extension (72°C, 30 s); and a final extension step (72°C, 5 min).

F1 and F2 fragments were digested with XbaI, separated by agarose gel electrophoresis, and purified by the use of a Wizard PCR cleanup kit (Promega). Fragments were ligated with T4 DNA ligase, and the product was amplified by PCR using forward primer F1 (5′-TAGACTTCTGGGGCAAGGTC-3′) and reverse primer F2 (5′-GGTACGGAGATTGTCGCCC-3′) with GoTaq Green Master mix (Promega), under conditions of initial denaturation (95°C, 30 s); 30 cycles of denaturation (95°C, 10 s), annealing (56°C, 30 s), and extension (72°C, 30 s); and a final extension step (72°C, 5 min). The purified ligated F1-to-F2 fragment was inserted into pGEM cloning vector (Promega), and the pGEM-F1F2 product was transformed chemically into E. coli strain DH5α. Positive transformants were selected on LB agar plates containing 0.1 mg/ml ampicillin, 80 μg/ml 5-bromo-4-chloro-3-indolyl-β-d-galactopyranoside (X-Gal) and 0.5 M IPTG. Colony transformants were screened by PCR for insertion and correct orientation of the fragment using forward primer 5′-TAGACTTCTGGGGCAAGGTC-3′ and reverse primer 5′-GGTACGGAGATTGTCGCCC-3′ with Go-*Taq* Green Master mix (Promega) under the same PCR conditions as those described above. Plasmid pGEM-F1F2 was extracted from selected colonies by the use of a Wizard MiniPrep kit (Promega), digested with XbaI, and treated with alkaline phosphatase. The Kan cassette PCR product was similarly digested, and the two digested products were ligated together overnight at 4°C, with a ratio of Kan cassette to pGEM-F1F2 of 7:1. Ligated product was transformed chemically into E. coli DH5α, and transformants were selected on LB agar plates containing 0.1 mg/ml ampicillin. PCR colony screening performed as described above was used to confirm the correct orientation of the insertion of F1-Kan-F2 (∼1,800 bp).

N. gonorrhoeae was transformed with a pGEM-F1-Kan-F2 construct as described previously (14). Briefly, gonococci were grown overnight on GC agar plates and a suspension of ∼1 × 10^8^ CFU bacteria was made in 1 ml of supplemented GC broth containing 5 mM MgCl_2_. A 200-μl volume of a bacterial suspension was added into the wells of a 24-well tissue culture plate (Greiner Bio-One) with ∼1,000 ng/μl of PCR product or plasmid. The plate was incubated for 20 to 30 min at 37°C with 5% (vol/vol) CO_2_. Prewarmed supplemented GC broth (1.8 ml) was then added to each well, followed by incubation for 4 h at 37°C with 5% (vol/vol) CO_2_. A bacterial suspension (100 to 200 μl) from each well was plated onto selective GC agar plates followed by incubation for 24 to 48 h. Single colonies growing on these plates were grown on fresh selective GC agar plates, and positive transformants were evaluated by PCR for the presence or absence of the *ng-acp* gene as described above.

The presence or absence of Ng-ACP protein in mutants and complemented strains was confirmed by Western blotting of selected transformed bacteria probed with murine antiserum (1/100 dilution) to rNg-ACP-Al(OH)_3_ and detected with anti-mouse Ig-alkaline phosphatase conjugate (Bio-Rad) (1/3,000 dilution).

### Accession number(s).

The structure of the rNg-ACP protein has been deposited with the Protein Data Bank (PDB) under accession code 6GQ4.
